# High salt intake and bone health in postmenopausal women: exposing the lack of studies – a systematic review and meta-analysis

**DOI:** 10.3389/fendo.2025.1694539

**Published:** 2025-11-19

**Authors:** Dinara Imash, Arnur Gusmanov, Mei-Yen Chan

**Affiliations:** 1 Department of Biomedical Sciences, School of Medicine, Nazarbayev University, Astana, Kazakhstan; 2 School of Public Health, University of Pittsburgh, Pittsburgh, PA, United States

**Keywords:** sodium intake, high salt intake, dietary salt, urinary calcium excretion, calcium loss, bone health, postmenopausal women, osteoporosis risk

## Abstract

**Introduction:**

Osteoporosis is a common health concern among women after menopause, partially due to declining estrogen levels, which are a major regulator of bone metabolism and calcium retention. This work presents the first systematic synthesis of evidence assessing the impact of dietary sodium intake on bone health among postmenopausal women. Although there is substantial research demonstrating that reducing sodium intake can lower urinary calcium excretion, much of this evidence has been derived from studies conducted in mixed populations, often without stratifying by age, sex, or hormonal status. Postmenopausal women, who are at increased risk for bone loss and calcium imbalance due to hormonal changes, remain significantly underrepresented in this body of literature. This research examines the impact of dietary salt on maintaining bone tissue structure and relates it to calcium excretion through urine as a marker of bone demineralization.

**Methods:**

A systematic review and meta-analysis were conducted in accordance with the guidelines for reporting systematic reviews. Systematic searching of four major databases, i.e., PubMed, EMBASE, Web of Science, and the Cochrane Library, was done to find reports of randomized controlled trials estimating the influence of sodium intake on calcium excretion in urine and on bone health indicators. Meta-analysis was used to synthesize evidence to estimate the effect of sodium intake on calcium excretion.

**Results:**

Six articles out of an initial 596 made it into the stringent selection criteria. The meta-analysis revealed that high sodium diets significantly increased urinary excretion of calcium in women after menopause, with a pooled mean difference of 29.38 mg/day (95% CI: 12.31 to 46.46, p < 0.01). These findings highlight the detrimental impact of excessive salt consumption on bone health, demonstrating a link between sodium consumption and accelerated loss of bone mass. Conclusion: Based on the findings of the review, limiting dietary sodium consumption is recommended as a way to help prevent osteoporosis in postmenopausal women.

**Systematic Review Registration:**

https://www.crd.york.ac.uk/PROSPERO/view/CRD42025643910, identifier CRD42025643910.

## 1 Introduction

Bone loss is a natural, age-related process affecting both men and women, but in some individuals, it can progress to osteoporosis due to various risk factors (1, 2). Osteoporosis weakens the skeletal system by reducing bone mineral density (BMD) and impairing bone structure, thereby increasing the risk of fractures (3). Estrogen deficiency is a key contributor, especially in women, where the hormonal decline accelerates bone resorption, reduces skeletal integrity, and significantly raises the risk of fractures (4). While calcium and vitamin D are recognized for their importance in preventing osteoporosis (5), emerging evidence highlights the critical role of other dietary minerals, particularly sodium, potassium, magnesium, and phosphorus, in bone health. Long-term sodium intake above the recommended dietary allowance (RDA) results in excessive renal calcium loss, thus establishing a negative calcium balance, which increases bone resorption (6–8). This is known as the “calcium-sodium paradox” and is linked to the shared transport process of sodium and calcium in the renal tubules that creates a net negative calcium balance in a high-sodium diet (9).

In the study by Nordin et al. (10), high sodium intake caused relative sodium intake to result in more calcium loss in postmenopausal than premenopausal women. Experimental studies, such as that of Evans (11), have shown that postmenopausal women may be particularly susceptible to sodium-induced bone resorption. The analysis by Fatahi et al. (12) showed that in the > 50 age group, a salt-rich diet was significantly associated with an increased osteoporosis risk (OR = 1.20; 95% CI, 1.02–1.41). In the review by Cohen & Roe (13), authors cautioned that sodium alone would not meaningfully offset the risk of osteoporosis but that sodium-sensitive individuals, such as postmenopausal women, may benefit from lowered salt consumption.

Although markers like bone-specific alkaline phosphatase (BAP) and C-terminal telopeptide (CTX) reflect bone turnover, urinary calcium was selected for this study because it more directly and immediately reflects the impact of dietary sodium on calcium metabolism (14, 15).

Sodium and calcium metabolism are closely linked, as their reabsorption in the renal tubules is coupled: salt-rich diet impairs calcium reabsorption, increasing urinary calcium loss (11). This effect is particularly concerning in postmenopausal women, whose reduced estrogen levels diminish the kidneys’ ability to retain calcium (16). Consequently, estrogen-deficient women tend to lose more calcium for a given sodium load than their premenopausal counterparts (16).

This study offers the first comprehensive review of existing evidence on how dietary sodium influences bone health specifically in postmenopausal women. While numerous studies suggest that lower sodium intake reduces calcium loss through urine, much of this research has been conducted in heterogeneous populations, with limited attention to differences in age, sex, or hormonal status (10–12). As a result, postmenopausal women — who face a heightened risk (17, 18) of bone deterioration and calcium imbalance due to hormonal shifts — are largely overlooked. This review focuses on how salt consumption affects the preservation of bone structure and explores urinary calcium excretion as an indicator of bone mineral loss.

## 2 Materials and methods

We performed a systematic review and meta-analysis under the Preferred Reporting Items for Systematic Reviews and Meta-Analyses (PRISMA) checklist and guidelines (19). Our research question was created based on the PICO (Population, Intervention, Control, Outcome) concept. The study protocol was prospectively registered on PROSPERO Database (Registration ID: CRD42025643910). A thorough search strategy was used in four electronic databases: PubMed, EMBASE, Web of Science, and the Cochrane Library. Relevant studies were found by combining Medical Subject Headings (MeSH) phrases with keywords, including variations of ‘postmenopause’ OR ‘postmenopausal women’ OR ‘postmenopausal female’ AND ‘sodium’ OR ‘high-sodium’ OR ‘high sodium diet’ OR ‘salt’ OR ‘salt intake’ OR ‘high salt intake’ OR ‘sodium chloride’ OR ‘excess sodium’ AND ‘bone health’ OR ‘bone density’ OR ‘bone mineral density’ OR ‘calcium excretion’ OR ‘osteoporosis’ OR’’urinary calcium’ OR ‘bone resorption’ OR ‘calcium metabolism’ OR ‘bone turnover’. Boolean operators were employed to ensure broad coverage of eligible studies.

After removing studies that were duplicates or did not meet predetermined criteria, full-text evaluations were performed to determine their suitability for inclusion. The review incorporated randomized controlled trials, longitudinal cohort research, crossover studies, and case-control investigations examining how increased sodium consumption influences bone density among women after menopause.

### 2.1 Eligibility criteria

Eligible studies focused on healthy postmenopausal women or populations where women and men were analyzed separately. The interventions involved high sodium intake (≥2,000 mg/day) compared to the World Health Organization (WHO) recommended maximum for adults, which is less than 5g of salt per day. Studies reported outcomes such as markers of calcium metabolism (e.g., urinary calcium excretion) and bone mineral density (BMD) at sites like the spine, hip, and femoral neck). Articles published between 1980 and 2024 in English were included. Studies with interventions other than dietary or nutritional changes and those without specific data for postmenopausal women were excluded.

### 2.2 Data extraction

Data were extracted using a standardized extraction sheet to ensure accuracy and minimize bias. Collected data covered study details (authors, publication dates, and methodologies), participant demographics, sodium intake levels, outcome measures (e.g., urinary calcium excretion and BMD), and follow-up duration. Where necessary, data reported in non-standard units were converted for consistency. Data extraction differences were settled by discussion or by engaging a third reviewer.

Across the six included studies, dietary sodium intake was controlled through either fixed-menu metabolic diets or sodium chloride supplementation protocols ranging from 60 to 300 mmol Na/day, with intervention durations between 7 days and 5 weeks in crossover or randomized designs. All six studies used 24-hour urine collections, generally obtained on one to three consecutive days. Urine samples were acidified with 6 M HCl in Lietz et al. (18), collected with boric acid in Evans et al. (11), refrigerated without preservative in Zarkadas et al. (8), and not specified in Teucher et al. (6), Sellmeyer et al. (22), or Harrington et al. (23). Urinary sodium was measured by flame photometry or ion-selective electrode methods.

Urinary calcium was analyzed by atomic absorption spectrophotometry (8, 22, 23), colorimetric methods including the O-cresolphthalein complexone assay and KONE analyzer (11, 18), or isotope-dilution inductively coupled plasma mass spectrometry (ICP-MS) (6).

The risk of bias in the studies included was assessed using the Joanna Briggs Institute (JBI) Critical Appraisal Tool for Randomized Controlled Trials (20). The tool checks for selection, performance, detection, attrition, reporting, and other biases. In order to evaluate publication bias, we used both graph and statistical approaches. A funnel plot was used initially to assess asymmetry and subsequent statistical assessment by using Begg’s tests.

### 2.3 Statistical analysis

The statistical meta-analysis was conducted manually and using Stata/MP 18.09 (StataCorp LLC, Texas, USA). Data were extracted from studies that reported measurements as the mean ± standard deviation (SD) and the mean change ± SD for the pre-and post-intervention periods. When only the Standard Error of the Mean (SEM) was available, SDs were calculated using the formula SD = SEM × √N (21). All results obtained from the studies were converted to mg/day. The effect sizes were calculated using Mean Differences (MD) (21) between the treatment and control groups across each study. This method directly measures the average difference in urinary calcium excretion, presenting the results in the original units of measurement (mg).

### 2.4 Ethical considerations

Ethical approval was not needed for this systematic review and meta-analysis because it used previously published data without directly involving human participants or collecting new information.

## 3 Results

### 3.1 Study selection

**Figure 1:**
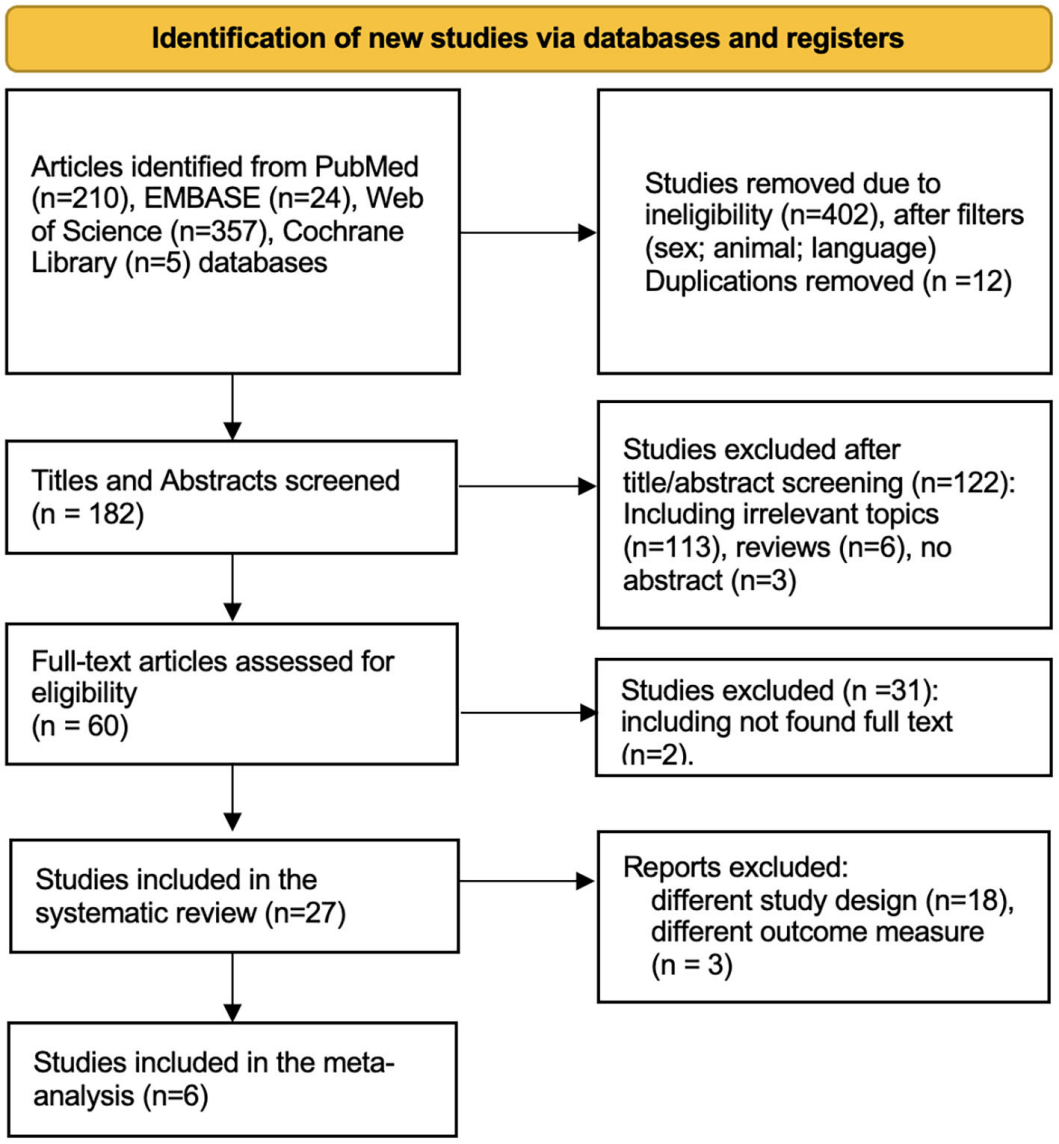
PRISMA diagram of study selection.

Subsequent application of exclusion criteria led to the removal of 402 studies for reasons such as inappropriate language (non-English), study type (animal studies, irrelevant time frame), and participant characteristics (non-human subjects). The PRISMA flow diagram summarizing this selection process is presented in **Figure 1**. After filtering, 182 titles and abstracts were screened, resulting in 122 being excluded due to their irrelevance to the research question. This left 60 full-text articles to be retrieved for more detailed examination. Of these, two full texts could not be retrieved, and an additional 29 did not meet the inclusion criteria based on their study designs, outcome measures, or relevance.

The 27 remaining studies underwent detailed evaluation to determine their suitability and quality. Of these, six studies fulfilled all inclusion requirements and were selected for the meta-analysis. Their primary characteristics are summarized below.

Sellmeyer et al. (22) and Teucher et al. (6) both studied high-sodium diets and how they affected bone health and the excretion of calcium through urine in women who had entered menopause. Sellmeyer et al. reported that potassium citrate supplements could effectively block the rise in urine calcium excretion that resulted from the high-sodium diet, whereas Teucher et al. found that moderately high sodium intake adversely affected bone balance when it accompanied high-calcium food intake. Bone resorption has been the focus of work by Evans et al. (11) and Harrington et al. (23); with increased resorption in postmenopausal women on high sodium diets being reported by (11) and (23) reporting the same effect based on vitamin D genotypes. Finally, increased sodium intake has been associated with a greater risk for osteoporosis (8) and (18) examined sodium effects on resorption markers and found little effect at levels in which they were studied.

### 3.2 Study characteristics

The meta-analysis assessed the impact of excessive dietary sodium intake on bone health in postmenopausal women through six randomized controlled trials. These trials directly evaluated the effects of different levels of sodium consumption on primary markers of bone metabolism, including urinary calcium, and indicators of bone resorption, including N-terminal Telopeptide and osteocalcin, in postmenopausal women. Some of the studies also evaluated urinary sodium, potassium, and phosphorus excretion.

A summary of the study participants is presented in **Table 1**. The mean age of the participants across the studies ranged from 57 to 63 years. All participants were healthy, postmenopausal women. The interventions varied considerably in duration, extending from as short as 5 days to as long as 35 days, assessing the effects of dietary sodium intake levels ranging from 102 mmol/day to 300 mmol/day.

**Table 1:** Characteristics of participants and intervention.

№	Author (year)	Study design	Population	Sample size (n)	Mean age	Intervention high sodium intake (mmol/d)	Intervention duration
1	Sellmeyer et al., 2002(22)	RCT	Postmenopausal women	26	63± 8	225	28 days
2	Zarkadas et al., 1989 (8)	RCT	Postmenopausal women	17	62.4 ± 1.3	102	5 days
3	Teucher et al., 2008 (6)	RCT	Postmenopausal women	11	59-73	192	35 days
4	Harrington et al., 2004 (23)	RCT	Postmenopausal women aged	24	57 ± 1	180	28 days
5	Evans et al., 1997 (11)	RCT	Postmenopausal women	11	57 ± 8.1	300	6 days
6	Lietz et al., 1997 (18)	RCT	Postmenopausal women	14	62 ± 3	170	8 days

### 3.3 Quality evaluation of included trials

**Figure 2:**
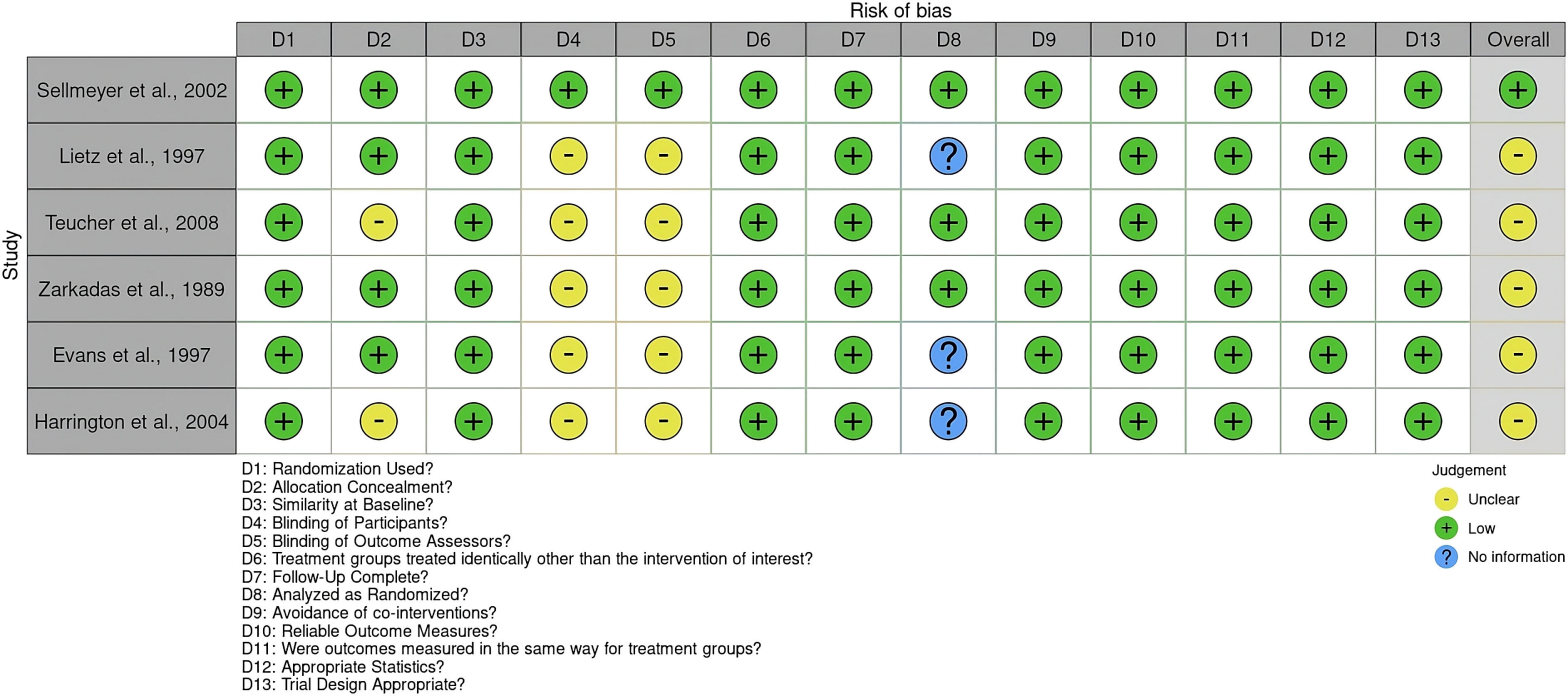
Risk of bias using the JBI critical appraisal checklist for randomized controlled Trials. The plot was created by RobVis software.

Two independent reviewers participated in the review process to ensure the accuracy and objectivity of the study selection. The quality of the included studies was evaluated using the Joanna Briggs Institute (JBI) Critical Appraisal Checklist for Randomized Controlled Trials (RCTs) before the meta-analysis (20). A traffic light risk-of-bias plot was generated to visually summarize the assessment results using RobVis web software (24) (**Figure 2**).

Out of the RCTs considered, the study by Sellmeyer et al. (22) received a high-quality rating, indicating a minimal risk of bias across all essential domains. The remaining five studies (6, 8, 11, 18, 23) had a moderate chance of bias, mostly because of a lack of participants and assessor blinding, as well as missing information on intention-to-treat analysis.

### 3.4 Meta-analysis results

**Figure 3:**
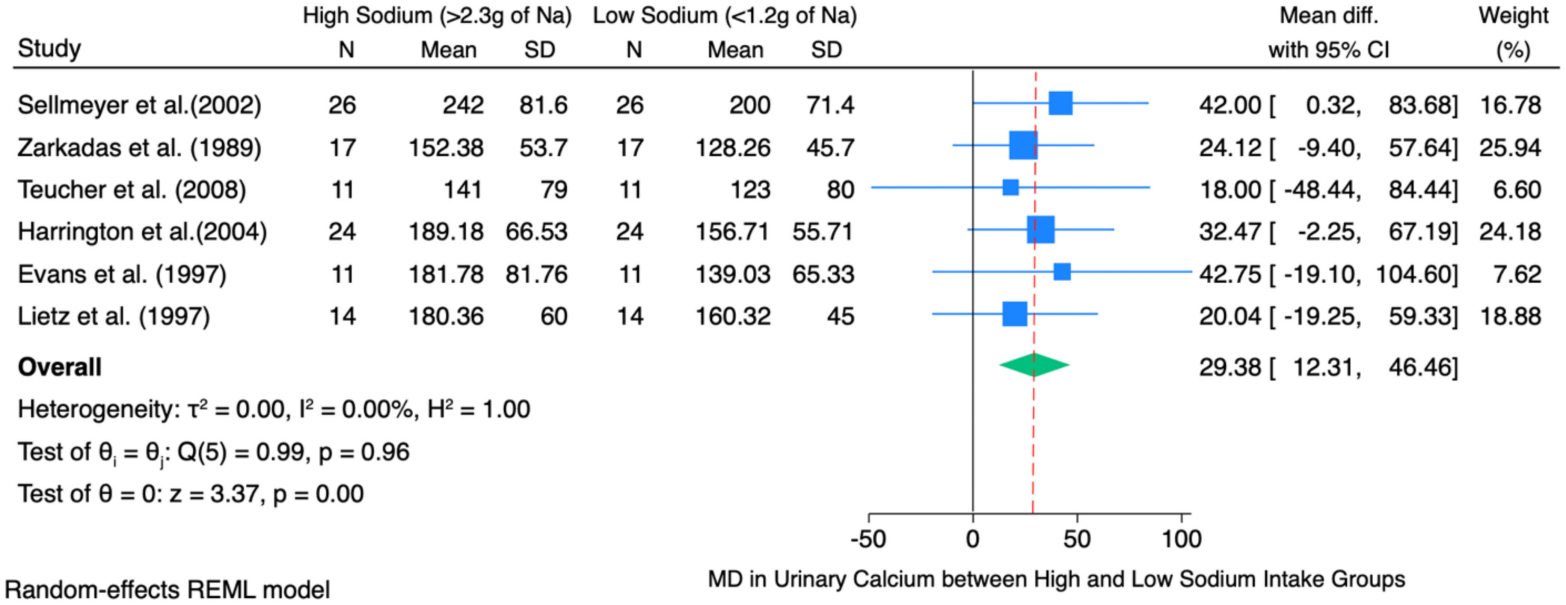
Forest plot of high sodium diet compared with low sodium diet groups showing difference in mean of change in 24-hour urinary calcium excretion.

**Figure 3** shows the pooled mean difference of 29.38 mg, which illustrates that, on average, a high sodium intake (>2.3 g Na/day) causes urinary calcium excretion to increase by about 29.38 mg as compared to a low sodium diet (<1.2 g Na/day) across six studies involving postmenopausal women. This finding is statistically significant, as evidenced by the 95% confidence interval ranging from 12.31 mg to 46.46 mg, and the z-value of 3.37 with a p<0.01. It was evident in the findings that increased sodium consumption was significantly related to increased urinary calcium compared to low sodium consumption. Raw mean differences were used to observe the average difference in urinary calcium excretion (in mg) between the high-sodium diet and the low-sodium diet across the included studies.

The individual study-level effects varied, with mean differences ranging from 18.00 mg/day (6) to 42.75 mg/day (11). However, most individual studies’ confidence intervals included zero, signifying that the observed differences at the individual study level did not reach statistical significance independently. Nevertheless, the pooled effect size demonstrated statistical significance and robustness.

The heterogeneity test indicated no significant heterogeneity among the studies (τ² = 0.00; I² = 0.00%; Q (5) = 0.99; p = 0.96). This suggests that the observed variations between studies are attributable to random chance rather than methodological or sample differences.

### 3.5 Publication bias

The evaluation of publication bias comprised the use of Begg’s test and funnel plot visual examination. Despite having only six studies (n=6), there was no evidence of significant publication bias (Begg’s test: z = -1.50, p = 0.2597). The funnel plot, in addition, confirmed there to be no asymmetry and thus an unbiased distribution of the published data (**Appendix 1**).

## 4 Discussion

This is the first systematic review investigating the effect of sodium intake on urinary calcium excretion, including a detailed meta-analysis of RCTs among postmenopausal women.

### 4.1 Interpretation of main findings

This systematic review and meta-analysis have established that excessive sodium intake is positively correlated with urinary calcium loss in postmenopausal women, substantiating the prediction that dietary sodium is an important, modifiable risk indicator for bone demineralization in such women. These findings consistently support the hypothesis that higher dietary sodium intake significantly contributes to greater urinary calcium excretion. This effect has important clinical implications, as sustained calcium loss via urine may negatively impact bone mineral density and increase osteoporosis risk, particularly among postmenopausal women.

Sodium intake affects calcium and bone metabolism through renal and systemic mechanisms (7, 9, 10, 16, 17). Sodium and calcium share transport pathways in the renal tubules; therefore, high sodium intake increases urinary calcium excretion by reducing tubular calcium reabsorption (9, 10, 16, 17, 36). This sodium-induced calciuria can disturb calcium balance and stimulate compensatory parathyroid hormone secretion, which increases bone resorption to maintain serum calcium levels (7, 17).

Studies in postmenopausal women have shown that habitual high sodium intake (≥ 3.5 g Na/day ≈ 9 g NaCl/day) leads to approximately 5–10% greater urinary calcium excretion and elevated bone-resorption markers such as C-telopeptide (CTX), N-telopeptide (NTX), and urinary deoxypyridinoline (DPD), which are associated with increased risk of developing osteoporosis (15, 17, 18, 22, 27).

On the other hand, it has been shown that diets high in fruit and green leafy vegetables will help to increase potassium intake which can help to reduce acid load and improve calcium retention (22, 27). Other supplements like calcium and vitamin D intake can offset the skeletal impact of sodium excess by maintaining calcium homeostasis and normal bone turnover (5, 27, 35).

Foods naturally rich in dietary calcium include milk, yogurt, cheese, and other dairy products, which provide highly bioavailable calcium and additional nutrients such as vitamin D, phosphorus, protein, and magnesium that contribute to bone formation and maintenance (34). Magnesium, essential for bone matrix formation and for the activation of vitamin D and regulation of calcium metabolism, is abundant in nuts, seeds, legumes, and whole grains (5, 33, 36, 37). In addition, regular consumption of vitamin D–fortified foods, such as fortified milk, breakfast cereals, and plant-based beverages, helps maintain optimal serum 25(OH)D concentrations, enhances intestinal calcium absorption, and supports skeletal health in postmenopausal women (27, 35, 37, 38). Collectively, these dietary and physiological mechanisms emphasize that the relationship between sodium intake and bone health is influenced not only by sodium-induced calciuria but also by the overall nutrient composition of the diet.

Within the systematic review and meta-analysis conducted by Fatahi et al. (12), scientists studied a wider population and utilized cohort and cross-sectional studies, comparing odds ratios to quantify osteoporosis risk, and correlation coefficients to measure associations with BMD. We validated their indications that increased sodium consumption substantially elevates osteoporosis risk, especially among postmenopausal women. The most recent cross-sectional study by Li et al. (25), evaluated the effects of low-sodium salt on BMD in the context of the Substitute Salt and Stroke Study. Findings showed lower prevalence rates and higher BMD in the low-sodium group by comparing osteoporosis and osteopenia rates between low-sodium and regular salt consumers. Their results suggest that low-sodium salt can be beneficial for bone health, especially among hyperglycemic populations.

These findings align with previous individual primary studies like Teucher et al. (6) and Zarkadas et al. (8), which reported that excessive sodium consumption was related to elevated calcium loss. The modest rise in calcium loss in our pooled effect size points to dietary modification as an important intervention strategy for preventing osteoporosis.

The lack of heterogeneity in the studies also reinforces the validity of our results, indicating that sodium’s effect on calcium excretion is uniform regardless of the minor differences in methodology. This uniformity is critical, given the worldwide increase in sodium intake, and it emphasizes the imperative of public health measures toward the prevention of this problem.

### 4.2 Evidence from meta-analyses and large-scale studies

Evidence from cohort studies is somewhat conflicting. A meta-analysis of observational studies by Fatahi et al. (12) found a link between high dietary sodium intake and an increased likelihood of osteoporosis, indicating higher odds of low bone density among individuals consuming more salt. Furthermore, a two-year randomized controlled trial conducted with postmenopausal women showed that decreasing sodium intake resulted in lower bone turnover, as indicated by biochemical markers (6). On the other hand, the large WHI observational analysis (N = 76,000) found no strong association between sodium intake (averaging ~ sodium 2.8 g/day) and fractures or BMD change (26), implying that within a common intake range, other factors (like calcium intake) modulate the effect. A Canadian cohort study also did not find a linear correlation between sodium excretion and BMD, though subgroup analysis suggested an effect in women with low calcium intakes (26). These discrepancies across large studies highlight heterogeneity: differences in baseline diets, range of sodium intake examined, and interactions with calcium/potassium intake can lead to varying outcomes (17).

Consistently, studies have indicated that getting sufficient calcium, vitamin D, and magnesium intake is necessary to support bone health, while the intake of sodium needs to be cut back to prevent bone density loss (27). Hormonal balance is also essential, and the protective role of estrogen in preventing bone loss in postmenopausal women is crucial.

### 4.3 Rationale for focusing on postmenopausal women

The choice of limiting this meta-analysis to postmenopausal women was motivated by the results from previous research on the high susceptibility of this group to osteoporosis (28). Prior studies have discussed that postmenopausal women are highly susceptible to enhanced calcium excretion due to estrogen deficiency, which disrupts bone metabolism and reduces the retention of calcium (29). Focusing on this group allows our study to fill a knowledge gap in the literature and provide targeted interventions that could guide specific dietary advice. Premenopausal women, who have normal hormonal control, do not suffer the same rate of calcium loss and, therefore, present a less useful group in the analysis of the direct correlation between salt consumption and the management of calcium (22).

### 4.4 Choice of urinary calcium excretion over bone turnover markers

Our analysis concentrated on urinary calcium excretion instead of other bone turnover markers. Due to the very specific focus of our sample (postmenopausal women), we encountered a significant limitation in gathering a sufficient number of studies that included bone turnover markers for our meta-analysis. In addition, numerous studies that assessed BMD were cross-sectional in design. Such a design is incompatible with the needs of our meta-analysis, which focused on randomized controlled trials. This method of measuring calcium excretion was in line with our aim of knowing the acute effects of sodium consumption on calcium balance. Nevertheless, it is important to acknowledge that the inclusion of both measures in studies in the future will also be useful to gain a more in-depth knowledge of the effects of sodium on bone health in the long term.

### 4.5 Limitations

This study is limited by the small number of studies that fit our inclusion criteria and differences in their years of publication. Included studies have been done between 1990 and 2010. Additionally, it is important to consider the racial backgrounds of participants, as Asian people are more prone to osteoporosis compared to white or other ethnic groups (30, 31). Despite that included trials in the analysis had participants of different races, race adjustments weren’t applied during the meta-analysis due to the small sample size. Besides, a high sodium diet is not the only risk factor for osteoporosis in postmenopausal women. Some of the other corresponding risk factors could be parental history (32), low activity levels (33), low calcium and Vitamin D (34, 35), smoking, and alcohol use.

### 4.6 Implications for practice and future research

Based on the findings, healthcare professionals should prioritize the restriction of dietary sodium as a measure of osteoporosis management among postmenopausal women. Health campaigns at the public level also need to aim at salt restriction programs as a protective measure against bone mineral loss, especially among Asian women.

Future studies need to follow up on these findings by examining the interaction of sodium and other minerals, such as calcium, potassium, and magnesium, and the combined effect on bone health. Further RCTs, which are specifically designed to measure both urinary calcium excretion and other bone turnover markers as well as change in BMD, will have to be conducted for the purpose of determining causality and measuring the longitudinal outcomes of sodium intake on bone health.

### 4.7 Conclusion

In conclusion, this systematic review and meta-analysis indicate that excessive salt intake correlates with elevated urinary calcium excretion and thus contributes to bone loss among postmenopausal women over time. The study emphasizes the need to consider dietary sodium limitation as an effective preventive intervention against osteoporosis in this high-risk population group. Additional research is required to develop dietary recommendations that could potentially lower osteoporotic risk in postmenopausal women.

## Data Availability

The data analyzed in this study is subject to the following licenses/restrictions: Requests to access these datasets should be directed to Not applicable.
